# Accuracy of a Novel SARS-CoV-2 Antigen-Detecting Rapid Diagnostic Test from Standardized Self-Collected Anterior Nasal Swabs

**DOI:** 10.3390/jcm10102099

**Published:** 2021-05-13

**Authors:** Bilgin Osmanodja, Klemens Budde, Daniel Zickler, Marcel G. Naik, Jörg Hofmann, Maximilian Gertler, Claudia Hülso, Heike Rössig, Philipp Horn, Joachim Seybold, Stephanie Lunow, Melanie Bothmann, Astrid Barrera-Pesek, Manuel Mayrdorfer

**Affiliations:** 1 Department of Nephrology and Medical Intensive Care, Charité–Universitätsmedizin Berlin, 10117 Berlin, Germany; klemens.budde@charite.de (K.B.); daniel.zickler@charite.de (D.Z.); marcel.naik@charite.de (M.G.N.); manuel.mayrdorfer@charite.de (M.M.); 2 Berlin Institute of Health, 10117 Berlin, Germany; 3 Institute of Virology, Charité–Universitätsmedizin Berlin, 13353 Berlin, Germany; joerg.hofmann@charite.de; 4 Labor Berlin–Charité Vivantes GmbH, 13353 Berlin, Germany; 5 Institute of Tropical Medicine and International Health, Charité–Universitätsmedizin Berlin, 13353 Berlin, Germany; maximilian.gertler@charite.de (M.G.); claudia.huelso@charite.de (C.H.); stephanie.lunow@charite.de (S.L.); melanie.bothmann@charite.de (M.B.); astrid.barrera-pesek@charite.de (A.B.-P.); 6 Medical Directorate, Charité–Universitätsmedizin Berlin, 10117 Berlin, Germany; heike.roessig@charite.de (H.R.); philipp.horn@charite.de (P.H.); joachim.seybold@charite.de (J.S.)

**Keywords:** COVID-19, COVID-19 diagnostic testing, SARS-CoV-2, SARS-COV-2 antigen testing

## Abstract

**Background** Antigen-detecting rapid diagnostic tests (Ag-RDT) for severe acute respiratory syndrome coronavirus 2 (SARS-CoV-2) offer new opportunities for the quick and laboratory-independent identification of infected individuals for control of the SARS-CoV-2 pandemic. Despite the potential benefits, nasopharyngeal sample collection is frequently perceived as uncomfortable by patients and requires trained healthcare personnel with protective equipment. Therefore, anterior nasal self-sampling is increasingly recognized as a valuable alternative. **Methods** We performed a prospective, single-center, point of care validation of an Ag-RDT using a polypropylene absorbent collector for standardized self-collected anterior nasal swabs. Real-time polymerase chain reaction (RT-PCR) from combined oropharyngeal/nasopharyngeal swabs served as a comparator. Primary endpoint was sensitivity of the standardized Ag-RDT in symptomatic patients with medium or high viral concentration (≥1 million RNA copies on RT-PCR for SARS-CoV-2). **Results** Between 12 February and 22 March 2021, 388 participants were enrolled. After exclusion of 9 patients for which no PCR result could be obtained, the novel Ag-RDT was evaluated based on 379 participants, of whom 273 were symptomatic and 106 asymptomatic. In 61 samples from symptomatic patients with medium or high viral load (≥1 million RNA copies), the sensitivity of the standardized Ag-RDT was 96.7% (59/61; 95% confidence interval (CI): 88.7–99.6%) for the primary endpoint. In total, 62 positive Ag-RDT results were detected out of 70 RT-PCR positive individuals, yielding an overall sensitivity of 88.6% (95% CI: 78.7–94.9%). Specificity was 99.7% (95% CI: 98.2–100%) in 309 RT-PCR negative individuals. **Conclusions** Here, we present a validation of a novel Ag-RDT with a standardized sampling process for anterior nasal self-collection, which meets World Health Organisation (WHO) criteria of ≥80% sensitivity and ≥97% specificity. Although less sensitive than RT-PCR, this assay could be beneficial due to its rapid results, ease of use, and suitability for standardized self-testing.

## 1. Introduction

Various antigen-detecting rapid diagnostic tests (Ag-RDTs) for severe acute respiratory syndrome coronavirus 2 (SARS-CoV-2) are now commercially available [1]. Performed in an appropriate way, they can support rapid decisions with respect to isolation, contact tracing, and treatment of patients with coronavirus disease 2019 (COVID-19) [2]. 

Since nasopharyngeal (NP) swabs are frequently perceived as uncomfortable by patients and must be collected by trained healthcare personnel, they are of limited use when establishing a population-wide testing strategy [3]. Fortunately, there is an increasing evidence base supporting the use of alternative sampling methods, including anterior nasal self-collection [4,5]. This easier sample collection method can aid to achieve higher reliability of Ag-RDTs for self-testing.

The primary objective of this prospective diagnostic accuracy study was to assess sensitivity and specificity for a novel Ag-RDT with supervised, self-collected anterior nasal swab sample using a porous polypropylene absorbent collector against the reference standard real-time polymerase chain reaction (RT-PCR) collected from an oropharyngeal (OP)/NP swab. 

## 2. Methods

The study protocol was approved by the ethical review committee of the federal state of Berlin and registered under ClinicalTrials.gov (NCT04698993). All experiments on human subjects were performed in accordance with the Declaration of Helsinki, implying that all participants provided informed consent. The study took place at two ambulatory SARS-CoV-2 testing facilities at Charité-Universitätsmedizin Berlin, Germany, from 12 February to 22 March 2021. At study site A, symptomatic adults suspected of SARS-CoV-2 infection were enrolled, while at study site B, asymptomatic and symptomatic employees and students were enrolled, participating in the regular hospital surveillance scheme. The main inclusion criterion for symptomatic patients was onset of COVID-19 symptoms within 7 days prior to testing. The main exclusion criteria were bleeding disorder, nasal spray application before testing, pregnancy and breastfeeding. Complete inclusion and exclusion criteria are provided in Table 1. Only participants with both an evaluable test result for the Ag-RDT and the RT-PCR reference standard were included in the analysis.

**Figure 1:**
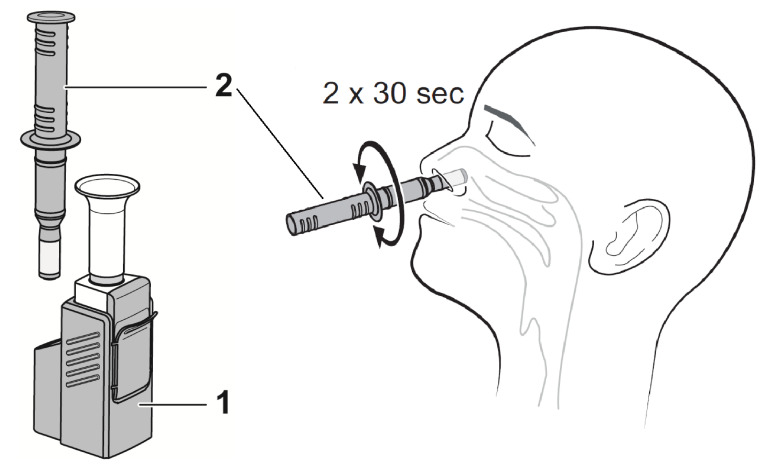
Sample collection-on the left, the test kit comprising a test cassette (**1**) and a removable sample collector (**2**) are shown. On the right, the sampling procedure is shown schematically.

First, participants received a combined OP/NP swab (eSwab from Copan with 1 mL Amies medium) as per institutional recommendations for RT-PCR. Subsequently, participants underwent an instructed, self-collected bilateral anterior nasal swab for the Ag-RDT (Dräger Antigen Test SARS-CoV-2 by Dräger Safety AG and Co. KGaA, Lübeck, Germany). The test comes as a one-piece test kit comprising a test cassette and a removable sample collector. The tip of the sample collector is a rigid, porous polypropylene sponge, which is used to swab the anterior nose and collect mucus and epithelial cells. After the general test procedure was explained to participants, verbal instruction was given to blow the nose once with a tissue. Next, the participants inserted the absorbent collector vertically 2–3 cm into the nostril and wiped the nasal walls in a circular motion for 30 s (Figure 1). This sampling process was repeated in the other nostril. After sampling, the sample collector was inserted and locked in the test cassette, where samples were analyzed immediately (within 15 min) after sampling at point-of-care by study physicians according to the manufacturer’s instructions. The test is designed as a sandwich immunoassay. Both capture antibodies and red-labelled detection antibodies (gold conjugate) bind SARS-CoV-2 nucleocapsid protein present in the sample, and a control line confirms that the assay was performed correctly. The test cassette is a self-contained unit, which allows sample analysis while avoiding further contact with potentially infectious material and making handling of additional liquids, e.g., pipetting or dropping buffers, unnecessary.

The Roche Cobas SARS-CoV-2 assay (Pleasanton, CA, USA) was performed on a cobas^®^ 6800/8800 analyzer (Roche Diagnostics, Mannheim, Germany) targeting both, *orf1a/b* (SARS-CoV-2) and *E*-gene (pan-Sarbecovirus). Viral concentration was classified into 3 categories (low: <1 million, medium: 1–10 million, high: >10 million RNA copies) based on standard preparations provided by Institute of Standardization, Düsseldorf, Germany. For all first-time diagnosed SARS-CoV-2 samples, typing for variants of concern (VoC) was performed, according to Phylogenetic Assignment of Named Global Outbreak (PANGO) Lineages classification [6].

Primary endpoint was sensitivity of the novel Ag-RDT in symptomatic patients with medium or high viral concentration (≥1 million RNA copies) on RT-PCR [7,8,9].

Secondary endpoints were overall sensitivity, sensitivity among patients with Ct-value ≤ 32, overall specificity, specificity in asymptomatic as well as symptomatic patients and frequency of nosebleed or unbearable pain due to specimen collection.

Data analysis was performed using the statistical programming language R, version 3.6.3 [10]. The R software package ggplot2 was used for data visualization [11]. Sensitivity and specificity were determined, and the corresponding confidence intervals were calculated using the Clopper–Pearson method. A two-proportions Z test was used for group comparison based on mutation status.

The study was stopped early, due to poor recruitment and Sponsor decision after 70 RT-PCR positive participants.

## 3. Results

**Figure 2:**
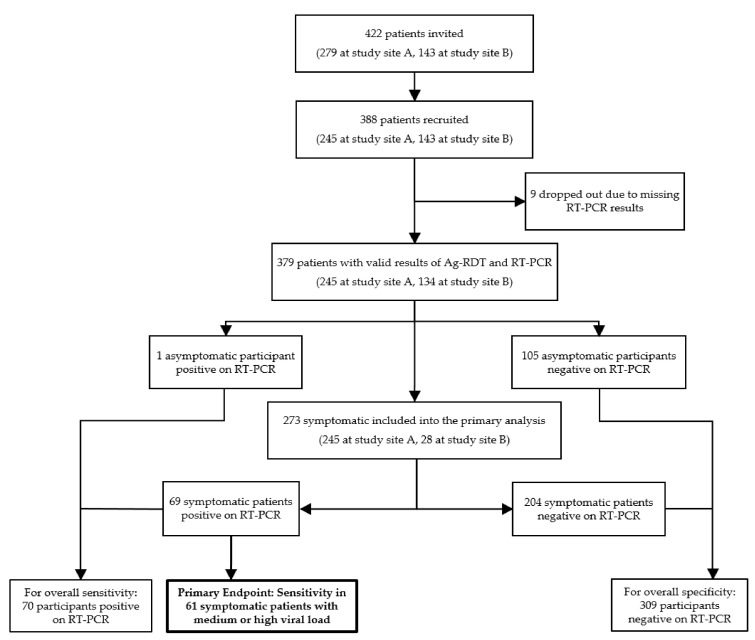
Participant flow diagram. RT-PCR: Real-time polymerase chain reaction; Ag-RDT: Antigen-detecting rapid diagnostic tests.

Of 422 patients invited, 388 (91.9%) consented to participate. Nine Ag-RDT negative participants (*n* = 7 asymptomatic, *n* = 2 symptomatic) were excluded as no RT-PCR result could be obtained until the end of the study due to data protection issues. For another symptomatic patient, RT-PCR found RNA at the limit of detection, but repeated RT-PCR testing was negative, hence the patient was classified as RT-PCR negative. In summary, 379 patients were included in the final analysis, of whom 273 were symptomatic and 106 were asymptomatic (Figure 2).

The average age of participants was 34.0 ± 10.8 years with 53.3% female and 46.7% male. Among all participants, 14.0% had comorbidities. Duration of symptoms at the time of presentation was on average 2.8 ± 1.8 days among the 273 symptomatic patients. Among all 379 participants, 70 (18.5%) tested positive for SARS-CoV-2 RNA, one of whom was asymptomatic.

### 3.1. Primary Endpoint

In 61 symptomatic participants with medium or high viral concentration (≥1 million RNA copies), the sensitivity of the Ag-RDT was 96.7% (59/61 RT-PCR positives detected; 95% confidence interval (CI) 88.7–99.6%). In 9 patients with low viral concentration (<1 million copies) only 3 tested positive with the Ag-RDT test (Table 2).

### 3.2. Secondary Endpoints

**Figure 3:**
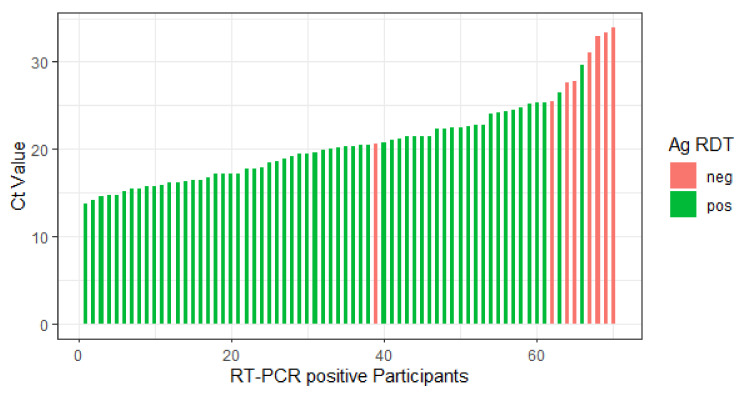
Bar plot showing Ag-RDT results and the corresponding Ct-values of 70 real-time polymerase chain reaction (RT-PCR) positive patients. Ct—Cycle threshold, ag RDT (antigen rapid diagnostic tests), neg—negative, pos—positive.

In total, the novel Ag-RDT showed a sensitivity of 88.6% (62/70 PCR positives detected; 95% CI 78.7–94.9%) as shown in Figure 3. Additionally, we analyzed sensitivity for patients with Ct-values ≤32, since this is another accepted threshold to discriminate high to significant from moderate to low viral concentration. Here, we found a sensitivity of 92.5% (62/67, 95% CI: 83.4–97.5%).

Overall, specificity was 99.7% (308/309 RNA negatives detected; 95% CI: 98.2–100%) compared to RT-PCR.

When regarding the 204 symptomatic patients who tested negative on RT-PCR, one tested positive with the Ag-RDT. Since the test line on Ag-RDT was very thin, the patient was symptomatic for 3 days, and PCR testing from combined OP/NP testing was reliably negative, we conclude that this showed a false positive result. Including this specimen, the specificity among symptomatic patients was 99.5% (203/204; 95% CI: 97.3–100%).

Among the 106 asymptomatic participants, one tested positive on RT-PCR with a Ct-value of 31, but was negative on Ag-RDT. For the remaining 105 asymptomatic participants, who tested negative on RT-PCR, specificity of the Ag-RDT was 100%.

Regarding mutation status, 44 participants were diagnosed with VoC B.1.1.7 and for the remaining 26 participants no VoC was found. Sensitivity of Ag-RDT among those patients with VoC B.1.1.7 was 88.6% (39/44) and did not differ significantly (*p* = 0.9075) from those without VoC, where sensitivity was 84.6% (22/26) [6].

### 3.3. Safety and Usability

During the study, no adverse events and no invalid results on Ag-RDT occurred. After a brief instruction, all patients were able to perform the test correctly under the supervision of the study physician. Regarding usability, comfort and safety, 4.2% (16/379 participants) reported light pain during the self-collection, but none reported strong or unbearable pain, and none developed nosebleed.

## 4. Conclusions

In this study, we demonstrated diagnostic accuracy of a novel Ag-RDT from self-collected anterior nasal swab, meeting the World Health Organisation (WHO) criteria of ≥80% sensitivity and ≥97% specificity [2]. Hereby, we follow other authors, who have demonstrated that supervised self-sampling from the anterior nose is a reliable option for Ag-RDT, yielding diagnostic accuracies comparable to those from nasopharyngeal swabs [4].

In comparison to other Ag-RDTs, the standardized absorbent collector used in this study is a rigid, porous sponge, which might reduce variability in sampling method. While Ag-RDTs are rather reliable, the Achilles heel of testing, and in particular of anterior nasal swabs, is sampling procedure. Most Ag-RDTs for self-testing rely on flexible specimen collectors that can be used to perform anterior nasal, mid-turbinate or nasopharyngeal sample collection. In contrast, using a standardized sampling procedure designed for anterior nasal testing may result in less variability, which is of utmost importance for the reliability of self-testing. Additionally, since the test comes as a self-contained device comprising all required biochemical reagents and the dilution buffer to carry out the test, the risk of contamination in the case of supervised self-testing is minimized for the testing personnel.

When compared to results from other test accuracy studies, the overall sensitivity of 88.6% found in our study is higher than that reported in a recent systematic review, where the average sensitivity of Ag-RDTs in the first week after symptom onset was 78.3% [12]. Among those patients with medium or high viral concentration, sensitivity in our study was 96.7%, which is comparable to the average sensitivity of 94.5% among patients with Ct values of ≤25 reported in the same review [12]. Among 106 asymptomatic participants, which we included in our study, only one tested positive on RT-PCR with a Ct-value of 31, but was negative on Ag-RDT. The remaining 105 participants correctly tested negative, which together with the overall specificity of 99.7%, makes positive results of this Ag-RDT highly reliable. One false-positive result on Ag-RDT occurred during the trial, which we attributed to unspecific binding to the test line. There were no uncertainties among the study physicians about the test result of the Ag-RDT and no invalid results, but weak test lines were counted as positive.

The differences in sensitivity emphasize the importance of patient selection for Ag-RDT. While Ag-RDT are of high sensitivity in the first week of disease, their sensitivity in the second week or among asymptomatic patients is only 51.0% or 58.1%, respectively [12]. There is an ongoing debate about the sensitivity of Ag-RDT in the very early symptomatic period, but our own data do not suffice to answer that question. We included 3 RNA positive participants on the first symptomatic day, all of which tested positive on both Ag-RDT and RT-PCR.

Limitations of our study arise due to the fact that OP/NP testing for RT-PCR was performed before Ag-RDT due to organizational reasons at the testing facility. In theory, this could transfer virus from the nasopharyngeal space to the anterior nose, but seems of little practical relevance since all patients were instructed to blow their nose to increase viral load in the anterior nose in any case. Another limitation in our and other studies is that Ct-values and viral concentration estimation are highly dependent on sample quality. In our study, experienced medical staff performed sampling and further processing was almost identical for all samples.

We used a cutoff of 1 million copies for the primary endpoint for two reasons. First, an internal standard of 1 million RNA copies was tested as a one-point calibration with each PCR run. Second, in addition to technical reasons, viral concentrations below 1 million RNA copies indicate a lack of contagiosity in the late phase of COVID-19, and can be used to guide isolation measures according to German healthcare authorities [7,8,9]. Since these cutoffs were validated in the beginning of the pandemic, it is conceivable that they have to be adapted for VoC, but to date the data available does not justify such decision.

Considering the ease of use of Ag-RDTs, self-sampling and patient self-testing is the main future use case for such tests. The standardized sampling and test procedure of the Ag-RDT investigated in this study may allow for more reliable self-testing, which can increase testing frequency and can have significant impact on the pandemic. It has received the CE mark and is currently commercially available for professional use.

## Figures and Tables

**Table 1:** Inclusion and exclusion criteria. General inclusion criteria apply for symptomatic as well as symptomatic participants, while specific inclusion criteria apply for the group stated. * Coronavirus disease 2019 (COVID-19) symptoms included fever, cough, sore throat, fatigue, general feeling of weakness, loss of smell or taste, shortness of breath, muscle stiffness, body aches, head cold, running nose, and others such as diarrhea or vomiting.

		Inclusion Criteria	Exclusion Criteria
**General**		≥18 years old Written informed consent Preexisting indication for Severe Acute Respiratory Syndrome Coronavirus 2 (SARS-CoV-2) testing, i.e., COVID-19 symptoms *, known or suspected exposure to SARS-CoV-2, screening	<18 years old Unable or unwilling to provide informed consent No preexisting indication for SARS-CoV-2 testing Pregnant or breast-feeding women Involuntarily held in an institution Bleeding disorder Application of nasal spray prior to testing on the day of testing Hospitalization/ inpatient treatment
**Specific**	Asymptomatic	Asymptomatic during the previous 14 days	COVID-19 symptoms during the previous 14 days
	Symptomatic	COVID-19 symptoms on the testing day Symptom onset 0–7 days prior to testing day	Symptom onset > 7 days prior to testing day

**Table 2:** Antigen-detecting rapid diagnostic test (RDT) results with a supervised self-collected anterior nasal swab in 70 RNA positive patients from combined oropharyngeal/nasopharyngeal swab. Abbreviations: Ct—cycle threshold, VC—Viral concentration, VoC—Variant of concern. Green—Positive antigen-detecting rapid diagnostic test. Red—Negative antigen-detecting rapid diagnostic test.

Ct (E-Gen)	VC-Copies	VoC	Days Symptomatic	Ct (E-Gen)	VC-Copies	VoC	Days Symptomatic
13.7	>10 Mio	B.1.1.7	4	20.3	>10 Mio	none	7
14.1	>10 Mio	B.1.1.7	1	20.4	>10 Mio	B.1.1.7	5
14.6	>10 Mio	B.1.1.7	3	20.5	>10 Mio	B.1.1.7	2
14.7	>10 Mio	none	2	20.7	>10 Mio	B.1.1.7	1
14.7	>10 Mio	B.1.1.7	1	20.8	>10 Mio	B.1.1.7	4
15.1	>10 Mio	B.1.1.7	1	21.1	>10 Mo	B.1.1.7	3
15.4	>10 Mio	B.1.1.7	3	21.2	>10 Mio	B.1.1.7	4
15.5	>10 Mio	B.1.1.7	3	21.4	>10 Mio	none	4
15.7	>10 Mio	B.1.1.7	3	21.4	>10 Mio	B.1.1.7	7
15.8	>10 Mio	none	2	21.5	>10 Mio	none	4
15.9	>10 Mio	B.1.1.7	1	21.5	>10 Mio	B.1.1.7	1
16.2	>10 Mio	B.1.1.7	2	22.4	>10 Mio	none	3
16.2	>10 Mio	B.1.1.7	3	22.4	>10 Mio	B.1.1.7	5
16.3	>10 Mio	none	1	22.5	>10 Mio	none	3
16.5	>10 Mio	none	4	22.5	>10 Mio	none	3
16.5	>10 Mio	none	3	22.6	>10 Mio	B.1.1.7	3
16.8	>10 Mio	none	6	22.7	1–10 Mio	none	3
17.1	>10 Mio	B.1.1.7	1	22.8	>10 Mio	none	0
17.1	>10 Mio	none	4	24.0	1–10 Mio	B.1.1.7	1
17.2	>10 Mio	B.1.1.7	1	24.2	1–10 Mio	B.1.1.7	2
17.2	>10 Mio	B.1.1.7	1	24.4	1–10 Mio	none	0
17.8	>10 Mio	B.1.1.7	1	24.5	1–10 Mio	B.1.1.7	3
17.8	>10 Mio	B.1.1.7	5	24.8	1–10 Mio	B.1.1.7	3
17.9	>10 Mio	B.1.1.7	6	25.2	1–10 Mio	B.1.1.7	7
18.4	>10 Mio	B.1.1.7	1	25.4	< 1 Mio	none	5
18.6	>10 Mio	B.1.1.7	6	25.4	1–10 Mio	none	7
18.9	>10 Mio	B.1.1.7	3	25.5	1–10 Mio	B.1.1.7	5
19.2	>10 Mio	B.1.1.7	1	26.5	<1 Mio	B.1.1.7	3
19.4	>10 Mio	B.1.1.7	2	27.7	<1 Mio	None26	2
19.5	>10 Mio	none	1	27.8	<1 Mio	B.1.1.7	3
19.6	>10 Mio	none	3	29.7	<1 Mio	none	0
19.9	>10 Mio	none	1	31.0	<1 Mio	none	asymptomatic
20.1	>10 Mio	B.1.1.7	2	33.0	<1 Mio	B.1.1.7	7
20.2	>10 Mio	B.1.1.7	5	33.3	<1 Mio	none	1
20.3	>10 Mio	B.1.1.7	3	33.9	<1 Mio	none	3

## Data Availability

De-identified data that underlie the results in this paper and analysis code are available until 5 years after publication to researchers who provide a sound proposal and all study sites and the Sponsor agree to sharing the data. Proposals should be directed towards the corresponding author.
